# How Does the (Re)Presentation of Instructions Influence Their Implementation?

**DOI:** 10.5334/joc.63

**Published:** 2019-04-18

**Authors:** Cai S. Longman, Baptist Liefooghe, Frederick Verbruggen

**Affiliations:** 1 University of the West of Scotland, Paisley, UK; 2 Ghent University, Ghent, BE

**Keywords:** Action and perception, Categorisation, Cognitive Control, Learning

## Abstract

Instructions are so effective that they can sometimes affect performance beyond the instructed context. Such ‘automatic’ effects of instructions (AEI) have received much interest recently. It has been argued that AEI are restricted to relatively simple and specific S-R tasks or action plans. The present study put this idea further to the test. In a series of experiments based on the NEXT paradigm (Meiran, Pereg, Kessler, Cole, & Braver, 2015a) we investigated the specificity of AEI. In Experiment 1, we presented category-response instructions instead of S-R instructions. Nevertheless, we observed AEI for novel stimuli from the instructed category (Experiment 1a), and abstractness of the category did not modulate the size of the NEXT effect (Experiment 1b). However, Experiment 2 revealed specificity at the response level: AEI were much smaller in conditions where the instructed GO response is semantically related to, but procedurally different from the required NEXT response, compared to a condition where the NEXT and GO responses were the same. Combined, these findings indicate that AEI can occur when S(C)-R instructions are abstract at the stimulus level, arguing against previous proposals. However, AEI does seem to require specificity at the response level. We discuss implications for recent theories of instruction-based learning and AEI.

## Introduction

Humans have the remarkable ability to quickly assimilate new tasks and to skilfully perform these tasks after a limited amount of practice. Chein and Schneider (2012) proposed that the proficient execution of new tasks is the result of three distinct learning stages. First, new behavioural ‘routines’ have to be created in a formation stage. This involves selecting and gating information from the perceptual and motor systems (Cole, Laurent, & Stocco, 2013) and chunking the relevant task components (Bhandari & Duncan, 2014); in other words, a ‘task-schema’ or ‘task-set’ is created. In a second step, this initial representation has to be implemented. At first, this involves cognitive-control routines, which oversee the implementation and make adjustments when required. Chein and Schneider referred to this as the ‘controlled-execution’ stage. Eventually, performance becomes automatic: people learn simple associations between stimuli and responses, reducing the need for further cognitive control (see also Logan, 1988; Shiffrin & Schneider, 1977). Whereas it was generally assumed that automaticity of behaviour requires practice, recent years have witnessed an exponential growth in studies indicating that automaticity can be attained without overt practice, but on the mere basis of instructions (see Brass, Liefooghe, Braem & De Houwer, 2017; Meiran, Liefooghe, & De Houwer, 2017 for reviews). In other words, automaticity of behaviour may occur even in the ‘controlled execution’ or early implementation stages.

The now prevailing view is that instructions, intended to be executed, can be implemented into a task-set, which guides their execution. Once a task-set is implemented, execution may not require much control any more; instead, instructions can have ‘automatic’ effects on behaviour (e.g., Brass, Liefooghe, Braem, & De Houwer, 2017; Cohen-Kdoshay & Meiran, 2007; Liefooghe, De Houwer, & Wenke, 2013; Liefooghe, Wenke, & De Houwer, 2012; Meiran, Cole, & Braver, 2012; Wenke, Gaschler, & Nattkemper, 2007; but see, Liefooghe & De Houwer, 2018). This idea is akin to Exner’s (1879) notion of the “prepared reflex” and that the implementation of instructions will lead to a state of preparedness. Meiran et al. (2012) referred to this phenomenon as *intention-based reflexivity*. The present study further scrutinized automatic effects of instructions (AEI) by determining the specificity of stimulus and response representations in an instructed task-set. Before we discuss our research question in more detail, we briefly introduce the paradigms used to study AEI, which will be at the core of the present endeavour.

### Paradigms used to study AEI

Inspired by prior research (e.g., Cohen-Kdoshay & Meiran, 2007; De Houwer, Beckers, Vandorpe, & Custers, 2005; Wenke, Gaschler, & Nattkemper, 2007), Liefooghe and colleagues (2012, 2013) introduced the *inducer-diagnostic paradigm* to study AEI. On each run, participants are presented with two tasks that share stimuli and responses: the inducer- and the diagnostic-task. First, the participant is presented with two new stimulus-response mappings relevant to the inducer task (e.g., X → left, Y → right). These instructions have to be maintained throughout the diagnostic task where the participant has to ignore the identity of the letter and respond only to its orientation (e.g., upright → left, italics → right). The inducer instructions are only relevant after the diagnostic task, when a stimulus from the inducer task is presented and participants have to execute one of the instructed S-R bindings. The colour of the stimulus indicates which task to perform (e.g., black → diagnostic (orientation) task, green → inducer (identity) task). Performance in the diagnostic task is better on response congruent trials (where the stimulus affords the same response in the diagnostic and inducer tasks) relative to incongruent trials (where the stimulus afforded different responses in each task). Liefooghe et al. (2012, 2013) labelled this difference the *instruction-based (task-rule) congruency effect*.

Although the inducer-diagnostic paradigm has its merits for investigating AEI, Meiran, Pereg, Kessler, Cole, and Braver (2015a) pointed out that the instruction-based congruency effect measured in the diagnostic task, may not be induced by instructions alone. Their rationale is that participants might accidentally apply the Stimulus-Response (S-R) mappings of the inducer task on trials of the diagnostic task. Following theories of automatization through overt practice (e.g., Logan, 1988, 1992), such task-misapplication may lead to the formation of S-R associations in long-term memory, which may in turn lead to a congruency effect. As a solution, Meiran et al. (2015a) introduced the ‘NEXT’ paradigm. Each mini-block again starts with the presentation of two S-R mappings (e.g., X → left, Y → right) that need to be applied in a 2-trial ‘GO’ phase. These instructions have to be maintained through a short ‘NEXT’ phase where the participant has to simply press one of the two response keys to progress through several ‘slides’ displaying one of the two instructed stimuli. The relevant phase is indicated by the colour of the stimulus (e.g., red = NEXT, green = GO). Even though no classification is necessary during the NEXT phase, response times are faster when the GO response associated with the stimulus is the same as the NEXT response (compatible) relative to when the required responses are different (incompatible). This difference is termed the *NEXT compatibility effect*.

In comparison to the inducer-diagnostic paradigm, the NEXT paradigm reduces the risk for task-misapplication because the NEXT-phase does not include a two-choice reaction time task as is the case for the diagnostic task. In addition, Meiran et al. (2015a) observed a reliable NEXT compatibility effect on the very first NEXT trial following the instructions (i.e., before the instructed rules could be practiced), thus completely ruling out the potential contribution of task practice. For these reasons, the present study used the NEXT paradigm.

### Specificity of the instructed stimulus and response

Several studies have observed that AEI are diminished under high working memory (WM) load conditions (e.g., Cohen-Kdoshay & Meiran, 2007; Meiran & Cohen-Kdoshay, 2012). This led to the conclusion that AEI are restricted to relatively simple S-R tasks or action plans (Cole, Braver, & Meiran, 2017; Meiran et al., 2017). When the retrieval of information has to occur in multiple steps (as is often the case for more complex action plans, including situations in which a large set of stimuli is mapped onto a specific response via their category membership), automatic execution of instructed responses may not occur. A study from Braem, Liefooghe, De Houwer, Brass, and Abrahamse (2017) provided some indirect support for this idea. They found that instructed S-R mappings produced AEI, but not the instructed task context (which can be considered to be a more abstract construct than a task stimulus). This could indicate that AEI are restricted to simple instructions that specify the S-R relationships.

The ‘specificity’ idea also receives support from the ‘implementation intention’ literature (see Martiny-Huenger, Martiny, & Gallowitzer, 2015, for a recent review). A large body of work indicates that goal attainment can be enhanced when people form simple action plans that link situations or cues in the environment to goal-directed actions (e.g., ‘If situation X arises, then I will do Y’). It has been argued that implementation intentions are effective because they allow the automatic execution of goal-directed actions. Importantly, in their review, Martiny-Huenger et al. argued that these plans have to be specific to be effective. For example, forming the intention to buy an apple (a specific item) in the canteen of your office building (a specific context) would be more effective than the intention to buy healthy food (a food category) for lunch (a vague context). In sum, it could be argued that instructions may have to be simple and specific to allow automatic implementation.

However, some recent research on practice-based learning and automaticity has questioned the relative importance of simple S-R associations (e.g., Hazeltine & Schumacher, 2015; Longman, Milton, Wills, & Verbruggen, 2018), and how a ‘stimulus’ and ‘response’ are represented (e.g., Henson, Eckstein, Waszak, Frings, & Horner, 2014; Horner & Henson, 2009, 2011; Longman, Kiesel, & Verbruggen, 2018). For example, Horner and Henson (2009, 2011) have demonstrated that at least two levels of stimulus representation (specific stimulus vs. abstract/semantic representation) can independently become associated with at least three levels of response representation (action, decision, classification). There is also some preliminary AEI evidence that instructions which link abstract stimulus and response codes can be implemented automatically. For example, Cohen-Kdoshay and Meiran (2007, 2009) investigated AEI for instructions describing category-response (C-R) bindings (e.g., first half of alphabet → left, last half of alphabet → right), suggesting that any stimulus from an instructed category might produce AEI (see also, Kiesel, Wendt, & Peters, 2007). Furthermore, using a variant of the inducer-diagnostic paradigm, Tibboel, Liefooghe, and De Houwer (2016; see also Tibboel & Liefooghe, in press) used words to describe items in the instruction phase, but images during the diagnostic phase. They also found AEI, indicating that semantic overlap may be sufficient. However, the words used in the instruction phase referred to very specific objects (e.g., ‘balcony’, ‘bomb’, ‘wall’), so it could be argued that the ‘specificity’ criterion was still met.

Similar findings have also been observed at the response level. Liefooghe et al. (2012) found AEIs even when the response effectors (e.g., index fingers on the left/right hand) or response modality (the spoken words ‘left’ and ‘right’) used in the diagnostic task did not match those used in the inducer task (e.g., middle fingers on the left/right hands). The latter suggests that the instructed response must also be represented at a more abstract level. This is consistent with previous research in the task-switching paradigm, which also observed task-rule congruency effects with responses that overlap only conceptually (e.g., Gade & Koch, 2005; Hübner & Druey, 2006; Schuch & Koch, 2003). Thus, even instructions that lack stimulus- or response specificity may elicit automatic effects.

In sum, the specificity of the response and stimulus representations included in task-sets induced on the basis of instructions remains unclear. On the one hand, some researchers propose that the automatic implementation of instructions is limited to instructions that specify the particular stimulus and action such that all relevant information (including specific exemplars) can easily be stored in WM (e.g., Cole et al., 2017; Martiny-Huenger et al., 2015). On the other hand, there is some preliminary evidence that AEI might also be found with more complex instructions that specify only categories of stimuli/responses and therefore rely on drawing additional information from longer-term memory stores. The available evidence, however, is dispersed across different studies and no systematic investigation has been conducted. Accordingly, the current experiments were designed to contrast AEI for instructions that varied in their degree of specificity, both at the stimulus (Experiment 1) and response (Experiment 2) level.

## Experiment 1

The aim of Experiment 1a was to determine whether instructed C-R mappings can be implemented automatically. Instructions specifying categories can be useful for large stimulus sets as they allow flexibility and generalizability across items or exemplars. However, a potential drawback is that it might not be possible to implement such instructions automatically. After all, when a specific stimulus is encountered, the category to which it belongs must be determined before the correct response can be selected. Thus, instructing C-R mappings may prevent automatic task implementation, and hence AEI.

To test this idea, we adapted the NEXT paradigm such that each item to be associated with a response had two exemplar stimuli. Experiment 1a consisted of two conditions. In one condition (*exemplar-based*), the instructions displayed both exemplar stimuli (drawings) from each item. In a second condition (*label-based*), the instructions displayed only the item names and not the specific exemplar stimuli used in the subsequent miniblock. By comparing the presence and magnitude of the NEXT effect between instruction conditions we could determine the relative impact of exemplar-based vs. label-based instructions on performance. If the NEXT compatibility effect is limited to the exemplar-based instructions condition, then we can conclude that only very simple (single-step) tasks can be implemented and executed automatically. On the other hand, if it is present in both instruction conditions then we can provide evidence in support of the notion that more abstract instructions are also implemented with a degree of automaticity.

Experiment 1a was limited to conditions where the instructions identified a specific item (e.g., ‘apple’) rather than a ‘category’ per se (e.g., ‘fruit’). In Experiment 1b, we introduced more abstract categories than those used in Experiment 1a and used only verbal labels in the instruction phase. In order to quantify the ‘abstractness’ of each category, we asked participants to rate each stimulus pair used in Experiments 1a and 1b on a scale from ‘highly concrete’ to ‘highly abstract’ and used this measure in the analyses to determine how levels of abstractness would influence AEI.

### Method

**Participants.** 80 students from the University of Exeter participated for £3 or partial course credits (Experiment 1a: *N* = 40 (35 female), age: *M* = 20.4 years, *SD* = 6.3; Experiment 1b: *N* = 40 (31 female), age: *M* = 23.5 years, *SD* = 8.9). The target sample size and exclusion criteria were decided in advance of data collection (when N = 40, we could detect small to medium-sized differences at 80% power in the omnibus ANOVA given an alpha of 0.05). For all experiments from the present study, we obtained approval by the local research ethics committee at the School of Psychology, University of Exeter, and participants provided written informed consent after the nature and possible consequences of the studies were explained to them.

**Apparatus and Materials.** Stimuli were presented on a 21.5-inch iMac using Psychtoolbox (Brainard, 1997). In both experiments, the stimuli themselves were black drawings of everyday items presented on a white background (the complete set of stimuli used in all experiments are available to download along with the Matlab scripts, raw data files and R scripts from the Open Science Framework data repository: https://osf.io/4hmpv/). The images were collected by performing free images searches on the internet. Each miniblock included two ‘categories’ or ‘stimulus sets’ (pseudorandomly selected from the entire set – e.g., swordfish, guitars) and each category/set included two exemplars. In Experiment 1b, the exemplar stimuli were grouped into more abstract categories (e.g., ‘fruit’) than those used in Experiment 1a, which could be defined according to a single item (e.g., ‘apple’). This manipulation was not systematic, and the categories/stimuli used in Experiment 1b were selected after testing for Experiment 1a was complete.

**Figure 1:**
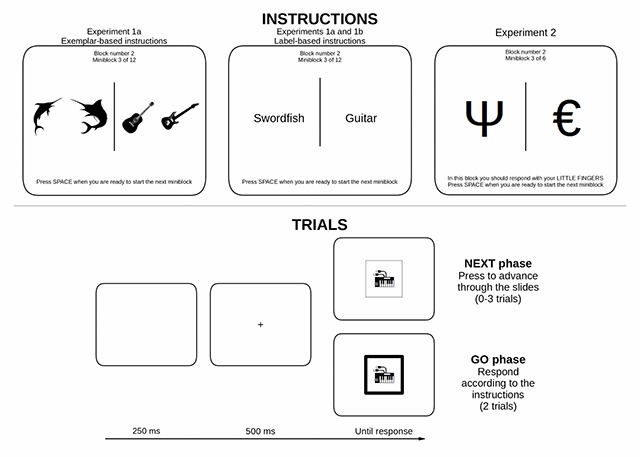
Miniblock instructions from each experiment (upper panel) and the timeline of a single trial during the NEXT and GO phases (lower panel) of each miniblock. In all experiments, the block number and miniblock number were presented at the top of the instructions screen which remained visible until the participant pressed the space bar (minimum 3 seconds). The additional instruction presented in Experiment 2 informed the participant which effector to respond with during the GO phase (little fingers in the example).

**The NEXT Procedure.** In both experiments, each miniblock was divided into three phases: the instruction phase, the NEXT phase, and the GO phase. Figure 1 shows some example instructions from each condition and also the time line of a single trial in the NEXT and GO phases.

The instructions phase differed depending on the condition and experiment. In the exemplar-based instructions condition of Experiment 1a, the four exemplar stimuli used in the miniblock were presented to the left and right of the centre of the screen (2 on the left and 2 on the right). In the label-based instructions condition of Experiment 1a and in Experiment 1b, only the names of the two items were presented to the left and right of the centre of the screen (black Arial font size 30). The location of each exemplar stimulus or label indicated the correct response for that exemplar/set (left vs. right button press – ‘a’ or ‘l’ keys respectively). The instructions were visible until the participant pressed the space bar (but remained visible for at least 3 seconds).

The NEXT phase lasted for 0, 1, 2, or 3 trials (the proportion of each run was 16.66%, 33.33%, 33.33%, and 16.66% respectively to partially control for participants anticipating the start of the GO phase based on the number of NEXT trials and to encourage preparation to perform the GO rules from the outset; see Verbruggen, McLaren, Pereg, & Meiran, 2018). During the NEXT phase, one exemplar stimulus (pseudorandomly selected to balance the number of compatible/incompatible stimuli presented on the 1^st^, 2^nd^ and 3^rd^ NEXT trial and with no immediate stimulus repetitions) was presented centrally surrounded by a thin black square (side = 50 mm; line thickness = 3 pt) to indicate that no categorization was necessary. Each NEXT trial started with a blank screen presented for 250 ms, followed by a fixation presented for 500 ms, after which the NEXT stimulus appeared. During the NEXT phase, the stimulus remained visible until the correct NEXT response was made (the total number of responses made was recorded for analysis). The NEXT response key (left or right) was counterbalanced over participants.

The GO phase started immediately after the NEXT phase ended and lasted for two trials only (stimuli were pseudo-randomly selected from the complete set of four exemplars used in the current miniblock, with no immediate stimulus repetitions either within the GO phase or from the last NEXT trial). The GO phase was identical to the NEXT phase except that the square surrounding the stimulus was bold (line thickness = 15 pt) to indicate that the stimulus should be classified according to the instructed rules. The time course of the GO trials was similar to the time course for NEXT trials, except that GO trials immediately ended when any response was made. The next miniblock started immediately after the second GO trial (in other words, there was no immediate feedback after individual miniblocks).

The experimental session of Experiment 1a consisted of 8 experimental blocks, including 12 miniblocks each (a total of 48 miniblocks per condition with a duration of 2–5 trials each; see above). Each block used only exemplar- or label-based instructions, and the format of instructions alternated on consecutive blocks (order counterbalanced over participants). The experimental session of Experiment 1b consisted of 4 experimental blocks, including 12 miniblocks each (a total of 48 miniblocks with a duration of 2–5 trials each). Mean GO performance (RT and accuracy) was presented at the end of each experimental block until the participant pressed the space bar to initiate the next experimental block.

Prior to the experimental session there was a brief familiarization phase. In Experiment 1a, this consisted of two practice blocks (three miniblocks per practice block, and each miniblock used novel stimuli). There was one practice block using exemplar-based instructions, and one using label-based instructions (order counterbalanced over participants). In order to provide the participant with some experience of the distribution of NEXT phase durations, each miniblock in each practice block used a different number of NEXT trials (0, 1 or 3; presented in a pseudorandom order). The familiarization phase of Experiment 1b consisted of only one practice block (consisting of three miniblocks). The data from the familiarization phases were not analyzed.

**Category ratings.** After completion of the experimental phase of Experiment 1b, the participants were asked to rate each stimulus pair. For completeness and as an extra manipulation check, we also asked them to rate the pairs from Experiment 1a. On each trial, we displayed the two exemplar stimuli from one pair (pseudorandomly selected) and the name of the category/item on the screen. The participant then had to rate the abstractness of the category by pressing one of the number keys (1–9) arranged across the top of a standard keyboard (1 = highly concrete, 9 = highly abstract). Participants were informed that a highly abstract category would be very general and could potentially include many examples from a wide range of sub-categories (e.g., ‘food’), whereas a highly concrete category would be very specific and might include very few examples with no obvious sub-categories (e.g., ‘Cox apples’).

**Dependent variables and Analyses.** All data processing and analyses were performed using R (R Core Team, 2017). In both experiments, the critical comparison was between NEXT performance on compatible trials (stimuli where the correct response during the GO phase was the same as the required response during the NEXT phase) vs. incompatible trials (stimuli where the correct response during the GO phase was different to the required response during the NEXT phase). Consistent with Verbruggen et al. (2018), we focused only on the first NEXT trial because AEI are largest on the first trial (Meiran et al., 2015) and performance on later NEXT trials could already be modulated by practice effects. Note that this decision was made prior to data collection, but it was important to include the remaining NEXT trials in the experiment to guarantee that the duration of the NEXT phase was unpredictable. This manipulation increases the likelihood that the instructions were maintained in a state that would allow their rapid implementation as soon as they became relevant.

Miniblocks where an incorrect response was made during the GO phase (Experiment 1a: 8.67%, Experiment 1b: 14.48%) were omitted from all NEXT analyses because this could indicate the instructions were not processed properly. We also excluded NEXT trials with RT <100 ms or >3000 ms resulting in a further 0.99% data loss in Experiment 1a, and 0.52% in Experiment 1b. The data from thirteen participants (Experiment 1a: 7, Experiment 1b: 6) were replaced because the above data cleaning procedures resulted in <10 (i.e., <50% of the maximum number of) observations per smallest cell (Instructions × Compatibility; see above). We decided on all selection and exclusion criteria before data collection had started.

Consistent with Verbruggen et al. (2018), we focused on three dependent variables. The primary dependent variable was the latency of the NEXT response (NEXT RT; equivalent to the analyses performed by Meiran et al., 2015). Because participants must press the NEXT key (see above) to progress through the NEXT phase, it is possible that NEXT RTs are slower on incompatible trials because participants press the incorrect key (i.e., the key associated with the presented stimulus) before pressing the NEXT key. We therefore also analysed the latency of the NEXT responses limited to those trials on which the first response was correct (Correct NEXT RT). Finally, we also analysed the proportion of errors made on NEXT trials (NEXT PE).

For Experiment 1a, we performed a separate Instructions (exemplar-based, label-based) by Compatibility (compatible, incompatible) ANOVA for each dependent variable. We also report Bayes factors and effect sizes (generalized eta squared) for all relevant effects/interactions. Bayes factors were calculated with the BayesFactor package, using the default JZS prior (.707; Morey, Rouder, & Jamil, 2015). To reduce the number of model comparisons, the interaction was only allowed if all constituent sub-effects were also included (see Morey et al., 2015). When this approach is used, Bayes factors <1 indicate that removing the effect/interaction from the full model is deleterious (i.e., it is a contributor to the fit of the full model). For all Bayesian analyses we report only the BF1 (i.e., the Bayes Factor for evidence in favour of the alternative hypothesis), and we interpret them using the classification discussed in Schönbrodt and Wagenmakers (2017). Finally, we performed paired-samples t-tests to directly assess the NEXT compatibility effect in each instructions condition. We applied a Holms-Bonferroni correction to correct for multiple comparisons. Bayes factors and effect sizes (Hedges’ *g*) are also reported for these analyses.

In Experiment 1b, we divided the categories into two groups (median split) based on the mean abstractness rating for each stimulus pair (concrete vs. abstract) and then performed Abstractness (concrete, abstract) by Compatibility (compatible, incompatible) ANOVAs on each dependent variable.^1^ Note that we used the average group ratings of each category to determine if a category was concrete or abstract (i.e., we did not use subject-specific ratings). Due to a technical issue, we had to run Experiment 1b twice. The results reported here are from the second version of the experiment (in which the issue was resolved). However, because the technical issue did not affect the collection of abstractness ratings, we used all of the available data when calculating the mean abstractness rating of each stimulus pair.

We also analysed the performance data from the GO trials in both experiments. As with the data from the NEXT trials, we first omitted any trials with RT <100 ms or >3000 ms from all analyses, resulting in 0.60% data loss in Experiment 1a and 1.12% in Experiment 1b. Error trials were also omitted from the GO RT analysis. We submitted the GO RTs and proportion of errors on GO trials (GO PE) to paired-samples t-tests (equivalent Bayes factors and effect sizes are also reported) to determine whether the instructions manipulation affected GO performance.

### Results and Discussion

**Figure 2:**
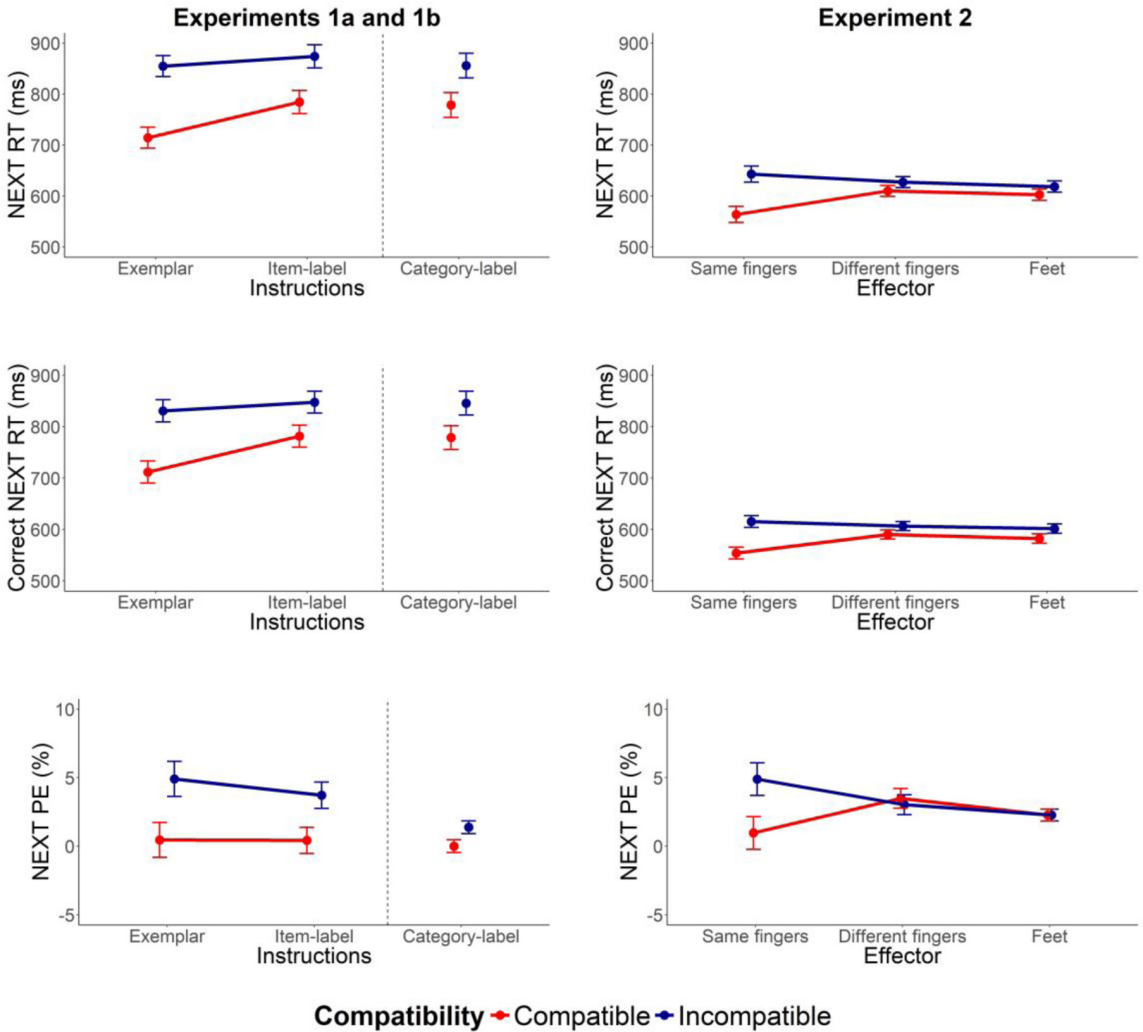
NEXT RTs (top), Correct NEXT RTs (middle) and NEXT PEs (bottom) from Experiments 1–2. Error bars show the between-subjects standard error of the mean difference between compatible and incompatible trials in each condition.

The mean NEXT RTs, Correct NEXT RTs and NEXT PEs are plotted in Figure 2 as a function of Instructions and Compatibility. The results from the ANOVAs are reported in Table 1 and the results from the t-tests are reported in Table 2.

**Table 1:** Omnibus ANOVA Results from Experiments 1a and 1b. Equivalent Bayes Factors are also Reported.

	NEXT RT					

Effect	*DF*	*MSE*	*F*	*p*	*BF*	*η^2^*

Experiment 1a						
Instructions	(1,39)	9,583.56	8.23	0.007	0.135 ± 3.3%	0.067
Compatibility	(1,39)	12,383.29	42.85	<0.001	<0.001 ± 3.3%	0.324
Compatibility × Instructions	(1,39)	6,357.96	4.05	0.051	1.368 ± 3.8%	0.023
Experiment 1b						
Abstractness	(1,39)	15,808.33	1.18	0.284	3.663 + 4.4%	0.008
Compatibility	(1,39)	26,505.30	6.53	0.015	0.092 + 5.6%	0.073
Compatibility × Abstractness	(1,39)	13,948.24	0.51	0.481	3.565 + 4.0%	0.003
	**Correct NEXT RT**					

**Effect**	***DF***	***MSE***	***F***	***p***	***BF***	***η^2^***

Experiment 1a						
Instructions	(1,39)	9,434.66	7.94	0.008	0.145 ± 5.8%	0.065
Compatibility	(1,39)	11,586.41	29.61	<0.001	<0.001 ± 6.7%	0.241
Compatibility × Instructions	(1,39)	6,713.58	4.19	0.047	1.089 ± 5.9%	0.025
Experiment 1b						
Abstractness	(1,39)	16,549.46	0.70	0.407	4.294 + 3.9%	0.005
Compatibility	(1,39)	24,205.70	5.17	0.029	0.247 + 5.5%	0.056
Compatibility × Abstractness	(1,39)	13,718.95	0.22	0.640	3.824 + 3.8%	0.001
	**NEXT PE**					

**Effect**	***DF***	***MSE***	***F***	***p***	***BF***	***η^2^***

Experiment 1a						
Instructions	(1,39)	15.08	1.00	0.322	4.552 ± 3.8%	0.006
Compatibility	(1,39)	37.00	16.25	<0.001	<0.001 ± 4.1%	0.190
Compatibility × Instructions	(1,39)	13.78	0.96	0.333	3.533 ± 2.7%	0.005
Experiment 1b						
Abstractness	(1,39)	9.07	1.99	0.166	1.981 + 4.0%	0.018
Compatibility	(1,39)	7.78	8.79	0.005	0.100 + 4.1%	0.063
Compatibility × Abstractness	(1,39)	9.07	1.99	0.166	1.570 + 3.9%	0.018

*Note:* Bayes factors indicate whether removal of the effect/interaction from the model would materially impair its fit. Thus, Bayes factors <1 indicate that the effect/interaction is an important contributor to the model.

**Table 2:** T-Test Results from Experiment 1a. Equivalent Bayes factors are also reported.

	NEXT RT							

	Difference	lower CI	upper CI	*DF*	*t*	*p*	*BF*	g_av_

Exemplar-based	141 ms	99	182	39	6.86	<**0.001**	414,283.458	0.834
Label-based	90 ms	44	136	39	3.95	<**0.001**	84.625	0.477
	**Correct NEXT RT**							

	**Difference**	**lower CI**	**upper CI**	***DF***	***t***	***p***	***BF***	**g_av_**

Exemplar-based	119 ms	76	163	39	5.54	<**0.001**	7,945.345	0.698
Label-based	66 ms	23	109	39	3.11	**0.004**	10.064	0.355
	**NEXT PE**							

	**Difference**	**lower CI**	**upper CI**	***DF***	***t***	***p***	***BF***	**g_av_**

Exemplar-based	4.5%	2	7	39	3.49	**0.001**	25.815	0.897
Label-based	3.3%	1	5	39	3.45	**0.001**	23.513	0.902

*Note:* p-values in bold font survived Holms-Bonferroni correction for multiple comparisons.

**Experiment 1a.** The ANOVA on the NEXT RTs found that participants were faster to respond on compatible (mean RT = 749 ± 24 ms) relative to incompatible trials (mean RT = 865 ± 32 ms; *p* < .001, *BF* < 0.001) – AEI. Participants also responded faster with exemplar-based instructions (mean RT = 785 ± 29 ms) relative to label-based instructions (mean RT = 829 ± 31 ms; *p* = .007, *BF* = 0.135) indicating that participants found the task easier when they had seen the stimuli prior to the start of the miniblock (even though the identity of the exemplars/items was irrelevant in the NEXT phase). The AEI were numerically larger in the exemplar-based instruction condition (mean difference = 141 ± 20 ms) than in the label-based instruction condition (mean difference = 90 ± 23 ms), but the Compatibility by Instructions interaction did not reach significance and the Bayesian analysis provided anecdotal evidence that removal of the interaction from the model would not materially impair its fit (Table 1). Critically, the planned contrasts found conclusive evidence of AEI or NEXT compatibility effects in both instruction conditions (see Table 2) indicating that AEI are not limited to conditions where the instructions display the relevant exemplar stimuli. As can be seen in Tables 1 and 2, the analyses on the Correct NEXT RTs and NEXT PEs broadly mirrored those of the NEXT RTs.

Finally, we correlated the NEXT compatibility effects from the two (within-subject) instruction conditions.^2^ We found positive correlations for the NEXT RTs (*r*(39) = .32, *p* = .042), Correct NEXT RTs (*r*(39) = .27, *p* = .097; though the correlation was not significant in this measure), and NEXT PEs (*r*(39) = .48, *p* = .002). These findings indicate that those participants with a large NEXT compatibility effect in one instructions format were also likely to have a large effect in the other – i.e., there was little evidence that the instructions manipulation resulted in large individual differences in performance across conditions.

Mean GO RTs and GO PEs are reported in Table 3. GO RTs were considerably faster in the exemplar-based instructions condition (mean GO RT = 689 ± 22 ms) than in the label-based instructions condition (mean GO RT = 735 ± 27 ms; *t*(39) = 4.06, *p* < 0.001, *BF* = 116.136, g_av_ = 0.295), indicating that the GO task was easier when the instructions specified the particular exemplar stimuli. GO PEs were broadly equivalent in both instructions conditions (GO PEs for exemplar-based instructions = 5.0 ± 0.6%; label-based instructions = 5.3 ± 0.6%; *t*(39) = 0.56, *p* = 0.576, *BF* = 0.198, g_av_ = 0.087).

**Table 3:** Descriptive Statistics for GO Performance in all Experiments. Standard Errors and 95% Confidence Intervals are also Reported.

		GO RT (ms)				GO PE (%)			

Experiment	Condition	Mean	SE	lower CI	upper CI	Mean	SE	lower CI	upper CI

Exp 1a	Exemplar-based	735	27	680	790	5.3	0.6	4.2	6.5
	Label-based	689	22	644	734	5.0	0.6	3.9	6.1
Exp 1b		875	38	798	953	9.1	0.8	7.4	10.8
Exp 2	Same fingers	568	15	538	599	8.1	0.7	6.7	9.5
	Different fingers	579	15	549	609	8.7	0.8	7.1	10.4
	Feet	630	18	594	665	7.0	0.6	5.7	8.3

**Experiment 1b.** NEXT RTs were faster on compatible NEXT trials (NEXT RT = 782 ± 35 ms) relative to incompatible NEXT trials (NEXT RT = 848 ± 38 ms, *p* = .015, *BF* = 0.092 ± 5.6%). However, neither the main effect of Abstractness nor the Compatibility by Abstractness interaction approached significance, and the Bayesian analyses provided moderate evidence that removal of the effect/interaction would not materially impair the fit (Table 1). Thus, AEI were not influenced by the abstractness of the instructed categories. More generally, this finding suggests that abstract C-R mappings can also be implemented automatically. Inspection of Table 1 shows that this pattern was observed for all dependent variables.

**GO phase.** Mean GO RTs and GO PEs are reported in Table 3. GO performance was broadly comparable whether the categories were more concrete (mean GO RT = 869 ± 40 ms, mean GO PE = 8.6 ± 0.9%) or more abstract (GO RT = 880 ± 37 ms, GO PE = 9.7 ± 1.0%). The small difference between categories did not approach significance for either dependent variable (GO RT: (*t*(39) = 0.88, *p* = 0.382, *BF* = 0.246, *g_av_* = 0.043; GO PE: (*t*(39) = 1.27, *p* = 0.212, *BF* = 0.359, *g_av_* = 0.180).

## Experiment 2

Experiments 1a and 1b focused on how the specificity of the instructed stimulus or category mappings influenced AEI, which are our marker of automatic instruction implementation. In Experiment 2, we turned our attention to the specificity of the instructed response codes. As described in the Introduction, Liefooghe et al. (2012) found in the inducer-diagnostic paradigm that AEI are robust to changes in response effectors and even response modalities; in other words, abstract response codes with conceptual overlap may produce AEI. The question was whether similar findings could be obtained in the NEXT paradigm. We ran a novel NEXT experiment in which the GO response was performed with either the index fingers, the little fingers, or the feet. The NEXT response was always a finger press. In the baseline (‘*same fingers*’) condition, the GO response was performed with the same fingers as the NEXT response (e.g., index fingers). In another (‘*different fingers*’) condition, the GO response was performed with different fingers to the NEXT response (e.g., NEXT response = index finger, GO response = little fingers). In the final (‘*feet*’) condition, the GO response was performed with the feet. If the NEXT compatibility effect is robust to these differences in the effectors used to respond in the NEXT and GO phases, then we can conclude that abstract response codes with conceptual overlap can indeed produce AEI.

### Method

48 different students from the University of Exeter (39 female) with a mean age of 20.8 years (*SD* = 3.2) participated for £7 or partial course credits. The number was higher than in the previous experiments due to the counterbalancing.

The apparatus, design and procedure were largely identical to Experiments 1a and 1b, but with the following differences. The stimuli were letters, numbers and symbols selected from readily available fonts (e.g., Wingdings, Webdings, Hebrew alphabet, ideograms, etc.), presented in black on a white background. Each miniblock included two stimuli (pseudorandomly selected from the entire set), one associated with a left response and the other associated with a right response (see Figure 1).

The set of relevant GO response effectors changed on every experimental block (but remained constant within each experimental block): left vs. right index fingers (‘f’ and ‘h’ keys respectively); left vs. right little fingers (‘a’ and ‘l’ keys respectively); left vs. right foot response (using a foot pedal response box). The NEXT response was always a manual response (left or right response with the index or little finger) and remained constant throughout the experiment (counterbalanced over participants). Whether the GO response used the same set of fingers (‘same fingers’ condition), different fingers (‘different fingers’ condition), or different extremities (‘feet’ condition) was included as a factor in our analyses. The experimental phase consisted of 48 experimental blocks of 6 miniblocks each (a total of 96 miniblocks per condition, each consisting of 2–5 trials). In each experimental block, the GO response was performed by one set of effectors (index fingers, little fingers, or feet). The relevant effector for each experimental block alternated throughout the experiment (e.g., index fingers… little fingers… feet… index fingers…) and the order was counterbalanced over participants. The maximum number of observations available for analysis in each effector condition was 40 compatible and 40 incompatible NEXT stimuli.

Prior to the experimental session there was a brief familiarization phase consisting of three practice blocks of three miniblocks each (each miniblock used novel stimuli), one practice block requiring index finger GO responses, one practice block requiring little finger GO responses, one practice block requiring foot GO responses (order counterbalanced over participants). Each miniblock in each experimental block of the familiarization phase used a different number of NEXT trials (0, 1 or 3; presented in a pseudorandom order). The data from the familiarization phase was not analysed. The entire session (including the familiarization phase) lasted approximately one hour.

The same exclusion criteria as in Experiments 1a and 1b resulted in the omission of 13.30% (incorrect GO responses) + 0.21% (data trimming) of the trials. In the GO phase, 0.32% of the correct RTs were removed after data trimming. The data from 9 participants were replaced because the number of observations per cell after data cleaning was too low.^3^

To analyze the NEXT data, we performed an Effector (same fingers, different fingers, feet) by Compatibility (compatible, incompatible) ANOVA for each dependent variable. The Effector by Compatibility interaction can reveal if AEI effects are influenced by specificity at the response level. We also performed paired-samples t-tests and their Bayesian equivalents to directly assess the NEXT compatibility effect in each Effector condition. We submitted the GO RTs and GO PEs to one-way ANOVAs with Effector as the within-subjects factor (equivalent Bayes factors and effect sizes are also reported) to determine whether GO performance was modulated by the effectors used to respond.

### Results and Discussion

**NEXT phase.** The mean NEXT RTs, Correct NEXT RTs and NEXT PEs are plotted in Figure 2 as a function of Effector and Compatibility. The results from the omnibus ANOVAs are reported in Table 4 and the results from the planned comparisons are reported in Table 5.

**Table 4:** Omnibus ANOVA Results from Experiment 2. Equivalent Bayes Factors are also Reported.

	NEXT RT					

Effect	*DF*	*MSE*	*F*	*p*	*BF*	*η^2^*

Effector	(2,94)	4380.65	1.478	0.233	6.364 + 5.4%	0.013
Compatibility	(1,47)	6236.84	16.28	<0.001	<0.001 + 4.0%	0.096
Effector*Compatibility	(2,94)	2681.63	11.5	<0.001	0.023 + 4.5%	0.061
	**Correct NEXT RT**					

**Effect**	***DF***	***MSE***	***F***	***p***	***BF***	***η^2^***

Effector	(2,94)	3516.26	1.484	0.232	5.213 + 2.2%	0.015
Compatibility	(1,47)	3779.39	19.755	<0.001	<0.001 + 2.7%	0.1
Effector*Compatibility	(2,94)	1705.54	8.371	<0.001	0.215 + 2.3%	0.041
	**NEXT PE**					

**Effect**	***DF***	***MSE***	***F***	***p***	***BF***	***η^2^***

Effector	(2,94)	9.81	2.558	0.083	5.002 + 2.6%	0.015
Compatibility	(1,47)	23.32	4.074	0.049	0.295 + 2.9%	0.028
Effector*Compatibility	(2,94)	13.43	10.446	<0.001	0.002 + 2.7%	0.079

*Note:* Bayes factors indicate whether removal of the effect/interaction from the model would materially impair its fit. Thus, Bayes factors <1 indicate that the effect/interaction is an important contributor to the model.

**Table 5:** T-Test Results from Experiment 2. Equivalent Bayes Factors are also Reported.

	NEXT RT							

Effector	Difference	lower CI	upper CI	*DF*	*t*	*p*	*BF*	g_av_

Same fingers	79 ms	47	111	47	5.03	<**0.001**	2,467.881	0.569
Different fingers	18 ms	–3	39	47	1.68	0.099	0.580	0.131
Feet	16 ms	–7	38	47	1.43	0.161	0.403	0.117
	**Correct NEXT RT**							

**Effector**	**Difference**	**lower CI**	**upper CI**	***DF***	***t***	***p***	***BF***	**g_av_**

Same fingers	60 ms	37	83	47	5.25	<**0.001**	4,970.890	0.476
Different fingers	17 ms	–1	34	47	1.87	0.067	0.782	0.131
Feet	20 ms	1	39	47	2.09	0.042	1.141	0.153
	**NEXT PE**							

**Effector**	**Difference**	**lower CI**	**upper CI**	***DF***	***t***	***p***	***BF***	**g_av_**

Same fingers	3.9%	1.5	6.3	47	3.30	**0.002**	16.823	0.751
Different fingers	–0.4%	–1.8	1.0	47	–0.63	0.534	0.189	0.112
Feet	0.0%	–0.9	0.8	47	–0.11	0.915	0.158	0.016

*Note:* p-values in bold font survived Holms-Bonferroni correction for multiple comparisons.

Participants responded faster on compatible (mean RT = 593 ± 19 ms) relative to incompatible trials (mean RT = 631 ± 20 ms; *p* < .001, *BF* < 0.001). The main effect of Effector did not reach significance (*p* = .233, *BF* = 6.364), but the difference in performance between compatible and incompatible stimuli during the NEXT phase *was* modulated by the effectors used to respond during the GO phase. Thus, in contrast to the findings of Liefooghe et al. (2012) and the current Experiment 1, specificity did seem to matter in Experiment 2. The NEXT compatibility effect was larger when the GO response was performed with the same fingers (mean difference = 79 ± 16 ms) relative to when the GO response was performed with different fingers (mean difference = 17 ± 11 ms) or with the feet (mean difference = 16 ± 11 ms; Effector by Compatibility interaction: *p* < .001, *BF* = 0.023). The planned contrasts found that the NEXT compatibility effect was reliable when the GO response was performed with the same fingers as the NEXT response (*t* = 5.03, *BF* > 1000). It did not reach significance when the GO response was performed with different fingers (*t* = 1.68, *BF* = 0.580) or with the feet (*t* = 1.43, *BF* = 0.403), but the Bayesian analyses in the latter two contrasts found only anecdotal evidence in support of the null hypothesis. Thus, even though we can conclude that AEI were substantially larger when the same effector was used and that specificity has an impact at the response level (the main aim of this experiment), further research is required to clarify whether the numerical differences in the expected direction for the ‘different fingers’ and ‘feet’ conditions represent abstract coding of instructed responses at all (cf. Liefooghe et al., 2012). Again, the analyses of correct NEXT RTs and NEXT PEs revealed similar patterns.

Taken together these results indicate that AEIs in the NEXT paradigm are largely limited to conditions when the GO and NEXT responses are executed with the same effectors (at best, AEI are markedly reduced when the NEXT and GO phases use different response effectors). This would seem to be at odds with Liefooghe and colleagues’ (2012) finding that the AEI in their paradigm were robust to changes in response effectors and even response modalities. There are at least three possible explanations for this apparent contradiction. First, it is possible that the frequent switching of response effectors in the current experiment might have induced shielding to reduce the response crosstalk between the two tasks. Second, response interference might be more likely to occur in two-choice tasks (in which multiple response options can be relevant), such as the diagnostic task of Liefooghe et al., compared to single-response tasks such as our NEXT task. Finally, it is possible that the generalization observed by Liefooghe et al. was the result of a practice effect – a possibility that we ruled out by only analysing performance on the first NEXT trial. We will come back to this issue in the General Discussion.

**GO Phase.** Mean GO RTs and GO PEs are reported in Table 3. GO RTs were fastest when the GO response was performed with the same fingers as the NEXT response (mean GO RT = 568 ± 15 ms), or with different fingers to the NEXT response (mean GO RT = 579 ± 15 ms) and was slowest when it required a foot response (mean GO RT = 630 ± 17 ms). The main effect of Effector was significant (*F*(2,94) = 25.69, *p* < 0.001, MSE = 1999.43, BF > 1000, η*^2^* = 0.353). The ANOVA on GO PEs found that participants made more errors when the GO response used the same fingers as the NEXT response (mean GO PE = 8.09 ± 0.7%) or different fingers to the NEXT response (mean GO PE = 8.72 ± 0.8%) than when the GO response was performed with the feet (mean GO PE = 6.99 ± 0.6%). This effect was also reliable (*F*(2,94) = 7.48, *p* < 0.001, *MSE* = 4.93, *BF* = 28.087 ± 0.6%, η*^2^* = 0.137), possibly indicating a speed-accuracy trade-off in GO performance – i.e., responding with the feet was slower, but more accurate than responding with the fingers.

## General Discussion

In two experiments, we examined the implementation of instructions in Meiran and colleagues’ (2015) NEXT paradigm. Specifically, we investigated how specific stimulus and response representations had to be in order to observe AEI. This question was motivated by some inconsistencies in the theories and findings in the existing AEI research. On the one hand, AEI are supposedly restricted to simple and specific instructions, which can be entirely represented within the limited capacity of working memory without the need to draw any additional information from longer term memory stores for their implementation (e.g., Cole et al., 2017; Meiran et al., 2012). On the other hand, some evidence suggests that AEI persist for more complex instructions that involve abstract representations (e.g., Liefooghe et al., 2012; Tibboel et al., 2016; Tibboel & Liefooghe, in press).

Experiments 1a and 1b focused on stimulus representations. In Experiment 1a, we contrasted a condition in which the GO stimuli (pictures) were shown during the instruction phase with a condition in which we used verbal labels (written text) to instruct the S-R mappings. We found a strong NEXT compatibility effect in both instruction conditions indicating that AEI can be triggered by novel (unseen) stimuli from the instructed category. The latter finding is consistent with Horner and Henson’s (2011) finding that S-R effects can be found when switching between text and picture stimuli. The similarity between studies suggests that instruction-based learning (as studied here) and practice-based learning (as studied in Horner & Henson, 2011) might have some features in common. It has been argued that both types of learning involve different routes. For example, Ramamoorthy and Verguts (2012) proposed that instruction following is implemented via a prefrontal (indirect) route that links stimuli and responses, whereas practice-based learning would be implemented via a (direct) route that includes stimulus areas, response areas, and the basal ganglia (see also Chein & Schneider, 2012). Importantly, a similar Hebbian learning mechanism might underlie both routes (Ramamoorthy & Verguts, 2012). Our findings seem consistent with this idea.

Experiment 1b was designed to determine whether AEI can also be found when the written instructions describe more abstract categories of stimuli. Thus, individual stimuli had to be mapped onto categories to determine the correct GO response. Interestingly, we still observed a reliable NEXT compatibility effect suggesting that novel stimuli from relatively abstract categories can trigger AEI. Furthermore, level of abstractness did not seem to modulate the NEXT effect much. These findings also indicate that AEI are not necessarily limited to very simple (single-step) tasks that do not compete for WM resources (cf. Cole et al., 2017). The multi-step process involved in classifying a stimulus according to its category membership before activating a response (it seems unlikely that participants could predict and retrieve the most likely exemplars from the more abstract categories and store all relevant information in WM) is rather more complex than the kind of processes previously thought to be the limit for AEI or the automatic activation of an implementation intention (cf. Martiny-Huenger et al., 2015). There are likely to be some pre-existing links between the items in the sets of stimuli/responses due to their semantic relationships; thus, the apparent transfer effects in Experiment 1b might be due to a combination of long-term stimulus-category associations and instruction-based C-R associations. It could be argued though that this combination is a standard feature of instruction-based learning. For example, Ramamoorthy and Verguts (2012) argued that tight associations between objects or attributes and their verbal labels are acquired during development, allowing the fast implementation of novel verbal instructions. A key principle of Cole, Laurent, and Stocco’s (2013) learning account is also that old representations can be reused in combination with a variety of other representations. Thus, the ability to follow and implement new instructions might be strongly rooted in the past (Verbruggen, McLaren, & Chambers, 2014).

Experiment 2 focused on response specificity. Previous studies suggest a response locus of AEI effects. For example, Meiran et al. (2015b) measured event-related potentials (ERPs) in a go/no-go version of the NEXT paradigm and found traces of activation of the incorrect (GO) response in the NEXT phase. This suggests unintentional activation of motor plans (see also Everaert, Theeuwes, Liefooghe, & De Houwer, 2014). Interestingly, we found that the NEXT effect was strongly modulated by the overlap in effectors. That is, the NEXT effect was weaker when the overlap between the GO and NEXT response was more ‘abstract’ (‘left response’ vs. ‘left index finger’). This contrasts with the findings of Experiment 1 in which we found little evidence that the NEXT effect was modulated by specificity/abstractness at the level of the stimulus.

The results of Experiment 2 would also appear to be in direct opposition to the results reported by Liefooghe and colleagues (2012) who found that the AEI were similar in the different effector conditions (if anything, the AEI effect was numerically largest in one of the conditions in which the inducer and diagnostic responses did *not* overlap). Because Liefooghe et al. analysed data from throughout the diagnostic task (equivalent to the NEXT phase), their result could potentially be explained by a practice effect (due to misapplication of the instructed task rules when they were not relevant), whereas we only analysed data from the first NEXT trial, ruling out this possibility. Another possibility is that the overlap between the tasks is greater in Liefooghe and colleagues’ procedure compared with the NEXT procedure used here. Importantly, both phases require a choice-selection in the Liefooghe et al. procedure, whereas the NEXT phase requires only a single response throughout. Task similarity might increase the probability of misapplication. A third related possibility is that response priming is constrained by the task set and how it is structured. Some work suggests that motor plans must be ‘prepared’ to a certain extent to allow automatic (unintentional) activation. For example, Verbruggen and Logan (2009) found that the task-irrelevant prime “STOP” only slowed responding in tasks in which subjects occasionally had to stop or withhold their response; when subjects were told that they could always respond, the prime did not influence performance. In other words, the stop response could only be activated when stopping was part of the task set (see also e.g., Folk, Remington, & Johnston, 1992, for similar findings in another domain). Structure of the task set might further modulate the priming effects or AEI. It has been argued that the different tasks in the NEXT or diagnostic/inducer procedures might become integrated into a single (hierarchical) task set in which the phase cue determines the correct response to the stimulus (e.g., Cole et al., 2017; Verbruggen et al., 2018). When a single task set is used, all response options might be prepared at the beginning of a block; and as a consequence, the various responses are more likely to be primed by task-irrelevant information. When the task components become more distinct (like in Experiment 2 of the present study), integrated task sets become less likely, reducing the probability that representations of the other task can be primed or activated. This ‘task separation’ could also explain the absence of response activation or priming effects in Experiment 2. Of course, our three explanations are post-hoc and more research is necessary to untangle this complex issue.

Because neutral trials (i.e., stimuli that were not associated with a particular response during the instructions phase) were not included in the NEXT phase of the current experiments, it is not possible to determine whether the NEXT effects reported here are due to compatibility-based benefits or incompatibility-based interference.^4^ However, Meiran et al. (2015a) included neutral NEXT trials in their Experiment 2 (using the same paradigm). They found that participants responded slower on incongruent NEXT trials than on neutral and congruent NEXT trials. However, the difference in performance between neutral and congruent NEXT trials was not significant. They concluded that intention-based reflexivity was therefore a result of incompatibility-related interference rather than a compatibility-related benefit to performance. It should be noted that Meiran et al. went to some lengths to ensure that their neutral condition was indeed neutral following some criticisms that it is very difficult to incorporate such trials in paradigms of this nature (e.g., Jonides & Mack, 1984). Our main aim was to investigate the degree to which the NEXT compatibility effect was dependent on specificity in the instructions rather than to investigate the source of the effect per se. Although we cannot entirely rule out the possibility that the introduction of more abstract instructions in the current experiments might have shifted the balance away from incompatibility-related interference toward compatibility-related benefits in Experiment 1, it seems unlikely that our manipulation would have had such a dramatic influence on the source of the NEXT effect. Similarly, it is not possible to determine precisely why participants found it easier to separate the tasks in the current Experiment 2. Nonetheless, the findings of Meiran et al. would suggest that the source of the separation was an increased ability to shield performance from incompatibility-related interference rather than compatibility-related benefits. Further research is required to conclusively answer these questions.

A potential criticism of the NEXT paradigm more generally is that interpreting the NEXT compatibility effect as evidence of AEI relies on the assumption that the NEXT stimulus is passively observed and not categorized according to the rules that are relevant for the GO task.^5^ In order to determine whether participants were (intentionally or unintentionally) practicing the GO task during the NEXT phase we performed several Pearson correlations comparing the NEXT compatibility effect with GO performance. If participants were practicing the GO task during the NEXT phase, then the size of the NEXT effect should be relatively large (because the tasks overlap in time), but latencies in the GO phase should be relatively small (because participants had a chance to practice the GO mappings in advance). Thus, a negative correlation between the NEXT effect and GO latencies should be observed. The correlations are reported in Table 6. Although none of the correlations were found to be significant, most were slightly positive (see Table 6). This pattern of results is inconsistent with the idea that participants had practiced the GO task during the NEXT phase. Note that Meiran, Pereg, Givon, Danieli, and Shahar (2016) have also observed a similar (positive) relationship between NEXT and GO performance.

**Table 6:** Results from the Correlations between the Absolute^6^ NEXT Compatibility Effect and GO performance in all Experiments.

Experiment	Condition	RT			PE		

		*DF*	*r*	*p*	*DF*	*r*	*p*

Exp 1a	Exemplar-based	39	0.07	0.672	39	0.10	0.520
	Label-based	39	0.04	0.797	39	0.20	0.210
Exp 1b		39	0.07	0.656	39	0.02	0.886
Exp 2	Same finger	47	0.03	0.826	47	0.12	0.400
	Different finger	47	–0.04	0.797	47	–0.19	0.190
	Feet	47	–0.16	0.288	47	0.11	0.466

*Note:* We used correct NEXT RTs for the latency analyses.

To conclude, the pattern of results reported here seem to be at odds with theories of AEI which claim they are limited to very simple tasks that can be entirely maintained in WM. Our findings also appear to be (at least partly) inconsistent with the implementation-intention literature, in which it has been argued that the automatic implementation of intentions (instructions) requires specificity (see e.g., Martiny-Huenger et al., 2015). We did observe specificity at the response level, but no (or at least less) specificity at a stimulus-level. Thus, the rapid and automatic influence of instructions on performance seems broader than initially thought.

## Data Accessibility Statement

All raw data files, R scripts (for data analysis), Matlab scripts (for data collection) and stimuli from all experiments are stored on the Open Science Framework data repository (https://osf.io/4hmpv/).

## Footnotes

As noted above, we asked all participants to rate the stimuli from Experiment 1a as well. As expected, participants rated the categories used in Experiment 1b as more abstract (mean abstractness rating = 5.43 ± 0.20) than those used in Experiment 1a (mean rating = 3.28 ± 0.18; *t*(114) = 21.21, *p* < 0.001, *BF* > 1000, *g_av_* = 1.771).

We thank an anonymous reviewer for suggesting these analyses.

In the Appendix, we report an analysis that included Experiment Half as a factor to examine how AEI evolved throughout the experiment. We replaced participants when there were fewer than 10 observations per smallest cell of the full design (i.e., Effector × Compatibility × 1^st^ vs. 2^nd^ half of the experiment).

We thank an anonymous reviewer for pointing this out.

We thank an anonymous reviewer for noting this.

We also ran the same correlations based on the proportional NEXT compatibility effect (e.g., (NEXT RT incompatible – NEXT RT compatible)/NEXT RT compatible) which also found little evidence for a relationship between NEXT performance and GO performance.

## Appendix: Session half analysis for Experiment 2

The duration of Experiment 2 was longer than Experiments 1a and 1b. Therefore, it was possible to also investigate the extent to which learning modulates the NEXT compatibility effect. Meiran and colleagues (2015) found some (albeit inconsistent) evidence that the NEXT compatibility effect reduces somewhat with learning, which was taken as evidence that AEIs are not subject to increased long-term memory automaticity as one might expect if (at least part of) it was skill-based. Verbruggen et al. (2018) replicated these findings and argued that subjects might learn about the overall task structure throughout the experiment, thereby making it easier to separate the GO phase from the NEXT phase (e.g., via hierarchical task structures). It was possible to investigate such learning effects in the present experiment by dividing it into equal halves and including this as a factor in the omnibus ANOVAs. The descriptive and inferential statistics appear in Tables A1 and A2 respectively.

**Table A1:** Descriptive Statistics for Experiment 2. Standard Errors and 95% Confidence Intervals are also Reported.

			NEXT RT (ms)					

Half	Effector	Compatibility	Mean	SE	lower CI	upper CI

1st	Same fingers	Compatible	606	19	568	645
		Incompatible	707	22	663	752
	Different fingers	Compatible	643	19	604	681
		Incompatible	673	20	632	713
	Feet	Compatible	653	23	607	700
		Incompatible	675	19	637	713
2nd	Same fingers	Compatible	522	20	481	562
		Incompatible	579	23	533	625
	Different fingers	Compatible	578	21	535	620
		Incompatible	582	23	536	628
	Feet	Compatible	552	22	508	596
		Incompatible	563	20	522	603
			**Correct NEXT RT (ms)**			

			**Mean**	**SE**	**lower CI**	**upper CI**

1st	Same fingers	Compatible	600	19	562	639
		Incompatible	675	20	635	715
	Different fingers	Compatible	626	18	589	662
		Incompatible	652	19	614	690
	Feet	Compatible	634	23	588	680
		Incompatible	657	19	619	695
2nd	Same fingers	Compatible	507	18	471	544
		Incompatible	555	20	515	596
	Different fingers	Compatible	555	19	516	593
		Incompatible	561	21	519	603
	Feet	Compatible	530	18	494	567
		Incompatible	546	19	508	584
			**NEXT PE (%)**			

			**Mean**	**SE**	**lower CI**	**upper CI**

1st	Same fingers	Compatible	0.7	0.3	0.1	1.3
		Incompatible	5.5	1.2	3.1	7.9
	Different fingers	Compatible	3.2	0.6	2.0	4.4
		Incompatible	3.9	0.8	2.3	5.6
	Feet	Compatible	2.3	0.5	1.3	3.3
		Incompatible	2.2	0.5	1.2	3.2
2nd	Same fingers	Compatible	1.2	0.6	0.0	2.4
		Incompatible	4.3	1.2	1.9	6.7
	Different fingers	Compatible	3.8	0.9	1.9	5.6
		Incompatible	2.1	0.6	0.9	3.3
	Feet	Compatible	2.2	0.6	1.0	3.5
		Incompatible	2.4	0.6	1.1	3.6

**Table A2:** Omnibus ANOVA Results from Experiment 2. Equivalent Bayes Factors are also Reported.

	NEXT RT					

Effect	*DF*	*MSE*	*F*	*p*	*BF*	*η^2^*

Half	(1,47)	13,939.84	97.20	<0.001	<0.001 ± 6.6%	0.283
Effector	(2,94)	8,771.23	1.26	0.289	9.760 ± 6.9%	0.006
Compatibility	(1,47)	12,551.15	16.12	<0.001	<0.001 ± 6.7%	0.056
Half × Effector	(2,94)	3,817.78	3.53	0.033	4.150 ± 7.4%	0.008
Half × Compatibility	(1,47)	3,274.07	7.75	0.008	1.359 ± 12.5%	0.007
Effector × Compatibility	(2,94)	5,347.06	11.74	<0.001	0.004 ± 7.0%	0.035
Half × Effector × Compatibility	(2,94)	3,731.67	0.85	0.430	9.070 ± 6.7%	0.002
	**Correct NEXT RT**					

**Effect**	***DF***	***MSE***	***F***	***p***	***BF***	***η^2^***

Half	(1,47)	12,384.41	112.23	<0.001	<0.001 ± 22.6%	0.347
Effector	(2,94)	6,904.48	1.32	0.272	7.025 ± 22.5%	0.007
Compatibility	(1,47)	7,327.39	20.53	<0.001	<0.001 ± 22.4%	0.054
Half × Effector	(2,94)	3,462.49	3.15	0.047	3.067 ± 22.7%	0.008
Half × Compatibility	(1,47)	2,766.86	4.28	0.044	1.951 ± 22.6%	0.004
Effector × Compatibility	(2,94)	3,304.81	9.27	<0.001	0.104 ± 26.7%	0.023
Half × Effector × Compatibility	(2,94)	2,969.63	0.38	0.686	10.022 ± 22.9%	0.001
	**NEXT PE**					

**Effect**	***DF***	***MSE***	***F***	***p***	***BF***	***η^2^***

Half	(1,47)	23.43	0.60	0.444	8.260 ± 5.4%	0.001
Effector	(2,94)	19.88	2.44	0.092	5.652 ± 10.8%	0.009
Compatibility	(1,47)	46.14	4.17	0.047	0.134 ± 6.5%	0.017
Half × Effector	(2,94)	12.60	0.49	0.612	22.484 ± 6.0%	0.001
Half × Compatibility	(1,47)	13.77	4.22	0.046	2.087 ± 3.9%	0.005
Effector × Compatibility	(2,94)	27.89	10.02	<0.001	<0.001 ± 43.0%	0.048
Half × Effector × Compatibility	(2,94)	15.73	1.33	0.270	6.295 ± 4.4%	0.004

*Note:* Bayes factors indicate whether removal of the effect/interaction from the model would materially impair its fit. Thus, Bayes factors <1 indicate that the effect/interaction is an important contributor to the model.

To avoid repetition of information reported in the main text, we focus on the main effect of Half and the interactions involving this factor. Participants responded faster during the 2^nd^ half of the experiment (mean RT = 563 ± 22 ms) relative to the 1^st^ half (mean RT = 660 ± 21 ms; main effect of Half: *F* = 97.20, *BF* < 0.001 ± 6.6%) suggesting that some learning had taken place during the experiment. The NEXT compatibility effect was slightly larger during the 1^st^ half of the experiment (mean difference = 51 ± 10 ms) than the 2^nd^ half (mean difference = 24 ± 11 ms; Half by Compatibility interaction: *F* = 7.75), which is consistent with the pattern of results reported by Meiran and colleagues (2015). However, the Bayesian analysis found anecdotal evidence that removal of the Half by Compatibility interaction from the model would not materially impair its fit (*BF* = 1.359 ± 12.5%). The Half by Effector interaction did reach significance (*F* = 3.53), but the Bayesian analysis found moderate evidence that removal of the interaction from the model would not materially impair its fit (*BF* = 4.150 ± 7.4%). The three-way interaction did not approach significance (*F* < 1, *BF* = 9.070 ± 6.7%). The analyses on the Correct NEXT RTs and NEXT PEs were again broadly consistent with the NEXT RTs (see Table A2).
